# Efficacy and safety of TACE combined with MWA followed by immune checkpoint inhibitors and anti-VEGF/tyrosine kinase inhibitors in hepatocellular carcinoma beyond the up-to-7

**DOI:** 10.3389/fimmu.2026.1885307

**Published:** 2026-07-28

**Authors:** Yang Fang, Jiangyu Tan, Xiaoting Su, Minghu Sun, Xianchun Zhou, Songnan Zhang

**Affiliations:** 1 Department of Oncology, the Affiliated Hospital of Yanbian University (Yanbian Hospital), Yanji, China; 2 Department of Interventional Radiology Center, the Affiliated Hospital of Yanbian University (Yanbian Hospital), Yanji, China; 3 Central Laboratory, the Affiliated Hospital of Yanbian University (Yanbian Hospital), Yanji, China

**Keywords:** hepatocellular carcinoma, microwave ablation, mRECIST, transarterial chemoembolization, UP-TO-7

## Abstract

**Background:**

Previous studies have shown that locoregional therapy combined with systemic treatment provides survival benefits with manageable safety in patients with intermediate-to-advanced large hepatocellular carcinoma (HCC). However, the optimal treatment strategy for HCC beyond the up-to-7 criteria remains unclear because of the high tumor burden and recurrence risk. This study aimed to evaluate the efficacy and safety of transarterial chemoembolization (TACE) plus microwave ablation (MWA) followed by targeted immunotherapy in patients with HCC beyond the up-to-7 criteria.

**Methods:**

This single-center retrospective study included patients with HCC beyond the up-to-7 criteria treated at our institution between October 2018 and December 2023. The study group received TACE plus MWA followed by ICIs combined with anti-VEGF/TKI therapy, while the control group received TACE followed by ICIs combined with anti-VEGF/TKI therapy. The primary endpoint was progression-free survival (PFS) assessed by mRECIST, and the secondary endpoints were overall survival (OS) and treatment-related adverse events (TRAEs).Baseline data were balanced using a one-to-one propensity score matching (PSM) and inverse probability of treatment weighting (IPTW).

**Result:**

Among the 80 included patients, the median follow-up time was 68.4 months. Thirty patients had hepatitis B and 40 had hepatitis C. Patients were classified as BCLC stage A–C, with a median tumor size of 7.1 cm. The control and study groups included 41 and 39 patients, respectively. The median PFS was 5.7 months(95%CI:3.617-7.716) versus 11.4 months(95%CI:8.741-14.125), and the median OS was 16.5 months(95%CI:10.863-22.071) versus 40.2 months (95%CI:34.302-46.165), respectively (both *P* < 0.05). After PSM analysis, the survival outcomes remained consistent with those observed in the primary cohort(both *P* < 0.05). Following sIPTW adjustment, the OS benefit remained statistically significant, whereas PFS showed a similar trend toward improvement(*P* = 0.051). Multivariate Cox analysis identified combination therapy as an independent protective factor for PFS and OS, while AFP ≥200 ng/mL was an independent risk factor for PFS. Most TRAEs were grade 1–2, with few grade 3–4.

**Conclusion:**

TACE combined with MWA followed by immune checkpoint inhibitors and anti-VEGF/TKI therapy prolonged progression-free and overall survival in patients with HCC beyond the up-to-7 criteria, with manageable safety.

## Introduction

Hepatocellular carcinoma (HCC) is the sixth most common malignancy worldwide and is characterized by high invasiveness and mortality (1). Owing to the lack of specific early symptoms, most patients are diagnosed at an advanced stage with high tumor burden or metastasis, thereby losing the opportunity for curative surgery (2). According to the Barcelona Clinic Liver Cancer (BCLC) staging system, transarterial chemoembolization (TACE) and systemic therapy are recommended standard treatments for intermediate-to-advanced HCC (3).Several large prospective studies, including EMERALD-1, CHANCE2005/CARES-005, and LEAP-012, have demonstrated the efficacy of TACE combined with targeted immunotherapy compared with TACE alone in unresectable intermediate-to-advanced HCC without extrahepatic metastasis (4–6), with progression-free survival (PFS) improved by up to 7 months (5). These findings have substantially reshaped the treatment landscape of HCC.HCC beyond the up-to-7 criteria, defined as the sum of tumor number and the diameter (cm) of the largest tumor exceeding 7 (7), is associated with high tumor burden and marked heterogeneity, leading to increased risks of recurrence and metastasis. Therefore, multimodal treatment strategies have become an important area of investigation.

More complete local tumor ablation may reduce recurrence and improve progression-free survival and overall survival (OS) (8). Microwave ablation (MWA) has shown efficacy comparable to surgical resection and may act synergistically with TACE to improve local control and outcomes (9–11). Meta-analyses have demonstrated that TACE combined with MWA is superior to TACE alone in large or recurrent tumors (≥5 cm) (9, 10).Although no standardized post-locoregional strategy exists, sequential immune checkpoint inhibitors (ICIs) plus anti-VEGF/tyrosine kinase inhibitors (TKIs) after local treatment may provide additional benefit (12). TACE and MWA enhance tumor antigen release and reshape the tumor immune microenvironment (13, 14), while anti-VEGF therapy inhibits angiogenesis and reduces recurrence (15). Small retrospective studies suggest that TACE plus MWA followed by targeted immunotherapy improves OS and PFS in early-to-intermediate HCC (16). However, evidence remains limited in patients with high tumor burden.

This study aimed to evaluate the efficacy and safety of TACE combined with MWA followed by immune checkpoint inhibitors and anti-VEGF/tyrosine kinase inhibitors in patients with HCC beyond the up-to-7 criteria, providing evidence for treatment strategies in patients with high tumor burden.

## Materials and methods

### Study population and inclusion/exclusion criteria

Between October 2018 and December 2023, we retrospectively analyzed clinical data from 118 patients with HCC beyond the up-to-7 criteria treated at Yanbian University Hospital. Patients received either TACE combined with MWA followed by ICIs plus anti-VEGF/TKIs (study group) or TACE followed by ICIs plus anti-VEGF/TKIs (control group). This single-center retrospective study was conducted in accordance with the Declaration of Helsinki (1975) and approved by the Ethics Committee of Yanbian University Hospital. Informed consent was waived due to the retrospective design. HCC was diagnosed according to the American Association for the Study of Liver Diseases (AASLD), the European Association for the Study of the Liver (EASL), or histopathological confirmation.

Inclusion criteria were: (1) age ≥18 years; (2) BCLC stage A–C; (3) beyond the up-to-7 criteria; (4) all patients received TACE, with up to one additional TACE allowed; patients in the study group additionally underwent MWA as an adjunctive locoregional treatment; (5) received ICIs plus anti-VEGF/TKIs within 1–2 months after completion of locoregional therapy; (6) liver function classified as Child-Pugh A–B or ALBI grade 1–2; (7) ECOG performance status 0; and (8) adequate hematologic function (WBC ≥3.0×10^9^/L, neutrophils ≥1.5×10^9^/L, platelets ≥75×10^9^/L, hemoglobin ≥85 g/L).

Exclusion criteria included prior liver transplantation or surgical resection; previous antitumor therapies including targeted therapy, immunotherapy, chemotherapy, radiofrequency ablation, cryoablation, or MWA; Child-Pugh C or ECOG ≥2; other malignancies; severe comorbidities such as infection or significant cardiac, pulmonary, or renal dysfunction; and incomplete clinical data.

The baseline adjustment factors were defined as follows: sex, age, hepatitis C virus (HCV) infection, cirrhosis, Child-Pugh grade, AFP level, tumor diameter, tumor number, BCLC stage, macrovascular invasion, and extrahepatic spread.

### Treatment regimen

All patients underwent TACE performed by experienced interventional radiologists. Doxorubicin was used as the chemotherapeutic agent, and iodized oil combined with embolic microspheres was used for arterial embolization to reduce tumor blood supply. A single TACE session was performed, with additional embolization allowed within 1 month for incompletely treated lesions.Two to four weeks after TACE, MWA was performed in the study group based on patient condition and liver function. The optimal puncture route was determined using ultrasound combined with CT guidance. Under general anesthesia, the microwave electrode was inserted toward the peripheral margin of the tumor, and CT imaging was used to confirm accurate positioning. Ablation power and duration were adjusted according to tumor size and location, with an intended ablation margin of 5–10 mm beyond the tumor edge. For larger lesions, incompletely embolized areas or tumors adjacent to high-risk intrahepatic locations were preferentially targeted for complete ablation. Post-ablation CT imaging was performed to confirm the ablation zone.

#### ICIs plus anti-VEGF antibody/TKIs administration

All patients received ICI plus anti-VEGF/TKI therapy within 1–2 months after completion of locoregional therapy (**Supplementary Table**). Dose reduction due to treatment-related adverse events (AEs) was permitted for TKIs but not for ICIs or bevacizumab (including its biosimilars). All agents were administered at standard doses and schedules. Dose reductions due to adverse events (AEs) were permitted for TKIs, whereas ICIs and bevacizumab or its biosimilars were not dose-reduced. Temporary treatment interruption due to AEs was allowed for both ICIs and anti-VEGF agents/TKIs. Systemic therapy was continued until disease progression or unacceptable toxicity.

#### Outcome measures and follow-up

The primary endpoint was progression-free survival (PFS), defined as the time from time zero (T0) to disease progression or death, whichever occurred first, T0 was defined as the initiation of ICI plus anti-VEGF/TKI therapy after eligibility criteria had been met. Secondary endpoints included overall survival (OS) and treatment-related adverse events (TRAEs). OS was defined as the time from T0 to death from any cause. Adverse events were graded according to the National Cancer Institute Common Terminology Criteria for Adverse Events (NCI-CTCAE) version 5.0.Follow-up was conducted through outpatient visits, telephone interviews, and imaging assessments. The final follow-up date was July 1, 2025. For patients lost to follow-up, the last date of confirmed contact was used as the censoring time.

### Statistical analysis

Statistical analyses were performed using SPSS version 29.0 and R software version 4.3.1. Continuous variables were presented as mean ± standard deviation or median (interquartile range) according to their distribution and were compared using the independent-samples t-test or Mann–Whitney U test. Categorical variables were expressed as numbers and percentages and compared using the chi-square test or Fisher’s exact test.Kaplan–Meier methods were used to generate progression-free survival (PFS) and overall survival (OS) curves, and differences between groups were compared using the log-rank test. Univariate and multivariate Cox proportional hazards regression analyses were performed to identify independent prognostic factors associated with PFS and OS, with hazard ratios (HRs) and 95% confidence intervals (CIs) calculated.Considering the potential treatment selection bias inherent to retrospective studies, propensity score matching (PSM) and stabilized inverse probability of treatment weighting (sIPTW) were performed as sensitivity analyses. PSM was conducted using 1:1 nearest-neighbor matching with a caliper width of 0.2. After sIPTW, the balance of baseline covariates between groups was assessed using standardized mean differences (SMDs), with an SMD <0.1 indicating adequate balance.The median follow-up duration was estimated using the reverse Kaplan–Meier method. All statistical tests were two-sided, and a *P* value <0.05 was considered statistically significant.

## Result

### Patient characteristics

**Figure 1:**
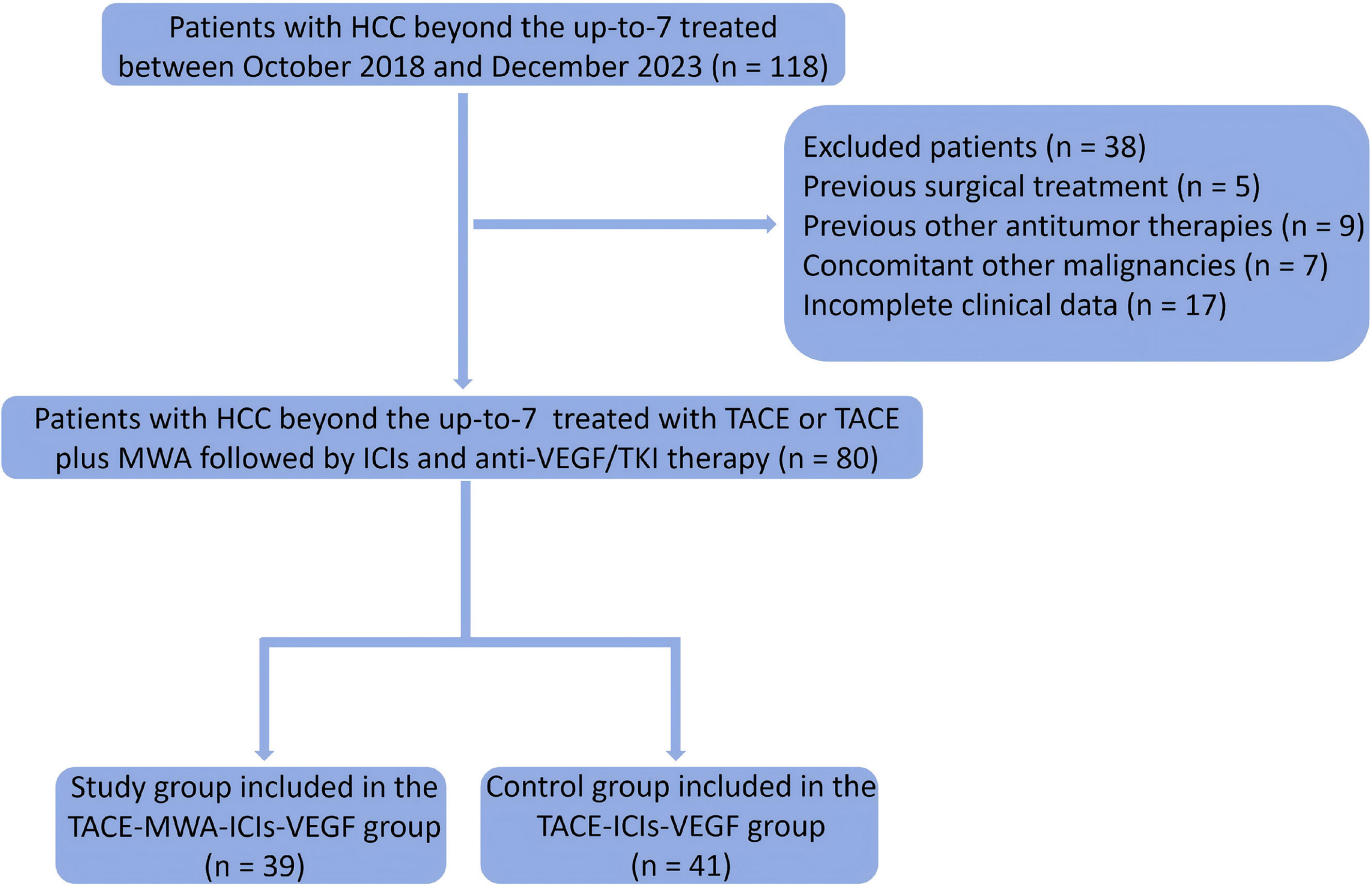
Flowchart of patient selection and grouping. HCC, hepatocellular carcinoma; TACE, transarterial chemoembolization; MWA, microwave ablation; ICIs, immune checkpoint inhibitors; VEGF, vascular endothelial growth factor.

All 80 patients were included in this study, with 39 in the study group and 41 in the control group(**Figure 1**). The median age was 66 years [interquartile range (IQR): 60–71], and 60 patients (75%) were male. Hepatitis B and hepatitis C were present in 30 (37.5%) and 40 (50%) patients, respectively. All patients were classified as BCLC stage A–C, with a median maximum tumor diameter of 7.1 cm. Non–main portal vein tumor thrombosis (PVTT) was observed in 7 patients, and 13 patients (16.25%) had extrahepatic metastasis. Baseline characteristics were well balanced between the two groups. To reduce potential selection bias, PSM and sIPTW sensitivity analyses were performed. Following PSM, baseline characteristics were generally well balanced between the two groups, although some covariates still showed slightly elevated SMD values above the predefined threshold. After sIPTW, all baseline covariates were well balanced between groups, with SMDs <0.1 (**Table 1**; **Supplementary Figure**).

**Table 1:** Baseline clinical data between the study group and contril group.

	Primary cohort				1:1 PSM cohort				sIPTW cohort		
Characteristics	Study group(n=39)	Control group(n=41)	*P* value	SMD	Study group(n=30)	Control group(n=30)	*P* value	SMD	Study group(n=38.1)	Control group(n=41.2)	SMD
**Age, years, Mean(SD)**	65.36(7.22)	65.20(9.01)	0.929	0.020	65.57 (7.61)	65.67 (9.79)	0.972	0.010	65.62(7.09)	65.49(9.22)	0.017
Sex, %											
Female	10(25.6)	10(24.4)	1.000	0.029	9(30.0)	8(26.7)	1.000	0.074	9.9(26.1)	11.1(26.9)	0.017
Male	29(74.4)	31(75.6)			21(70.0)	22(73.3)			28.2(73.9)	30.1(73.1)	
HCV, %											
Absent	22(56.4)	18(43.9)	0.371	0.252	16(53.3)	16(53.3)	1.000	<0.001	18.3(48.1)	19.7(48.0)	0.004
Present	17(43.6)	23(56.1)			14(46.7)	14(46.7)			19.8(51.9)	21.4(52.0)	
Cirrhosis, %											
Absent	20(51.3)	14(34.1)	0.186	0.352	13(43.3)	13(43.3)	1.000	<0.001	17.0(44.5)	17.3(42.1)	0.048
Present	19(48.7)	27(65.9)			17(56.7)	17(56.7)			21.2(55.5)	23.8(57.9)	
Child-Pugh class, %											
A	29(74.4)	24(58.5)	0.208	0.340	21(70.0)	19(63.3)	0.784	0.142	26.3(68.9)	27.6(67.1)	0.039
B	10(25.6)	17(41.5)			9(30.0)	11(36.7)			11.8(31.1)	13.5(32.9)	
AFP level, %											
<200ng/ml	27(69.2)	23(56.1)	0.326	0.274	18(60.0)	19(63.3)	1.000	0.069	25.4(66.7)	26.4(64.0)	0.057
≥200ng/ml	12(30.8)	18(43.9)			12(40.0)	11(36.7)			12.7(33.3)	14.8(36.0)	
Tumor Diameter, Mean(SD)	7.38(2.32)	7.98(3.14)	0.338	0.216	7.55(2.55)	7.14(2.30)	0.515	0.169	7.39(2.29)	7.57(2.83)	0.069
Tumor Number											
Single	18(46.2)	18(43.9)	1.000	0.045	15(50.0)	11(36.7)	0.434	0.272	17.6(46.3)	19.1(46.4)	0.002
Multiple	21(53.8)	23(56.1)			15(50.0)	19(63.3)			20.5(53.7)	22.(53.6)	
BCLC stage, %											
A	15(38.5)	13(31.7)	0.690	0.142	12(40.0)	9(30.0)	0.588	0.211	26.3(68.9)	27.6(67.1)	0.039
B/C	24(61.5)	28(68.3)			18(60.0)	21(70.0)			11.8(31.1)	13.5(32.9)	
Portal vein tumor thrombus											
Absent	35(89.7)	38(92.7)	0.945	0.104	27(90.0)	27(90.0)	1.000	<0.001	34.8(91.4)	37.9(92.0)	0.020
Present	4(10.3)	3(7.3)			3(10.0)	3(10.0)			3.3(8.6)	3.3(8.0)	
Extrahepatic metastasis											
Absent	34(87.2)	33(80.5)	0.612	0.183	26(86.7)	25(83.3)	1.000	0.093	31.9(83.6)	34.2(83.1)	0.012
Present	5(12.8)	8(19.5)			4(13.3)	5(16.7)			6.3(16.4)	6.9(16.9)	

Continuous variables are presented as mean ± standard deviation (SD), and categorical variables are presented as number (%). *P* values were calculated using the Student’s t-test or Mann–Whitney U test for continuous variables and the chi-square test or Fisher’s exact test for categorical variables, as appropriate. BCLC, Barcelona Clinic Liver Cancer.

PSM, propensity score matching; sIPTW, stabilized inverse probability of treatment weighting.

### Survival analysis

**Figure 2:**
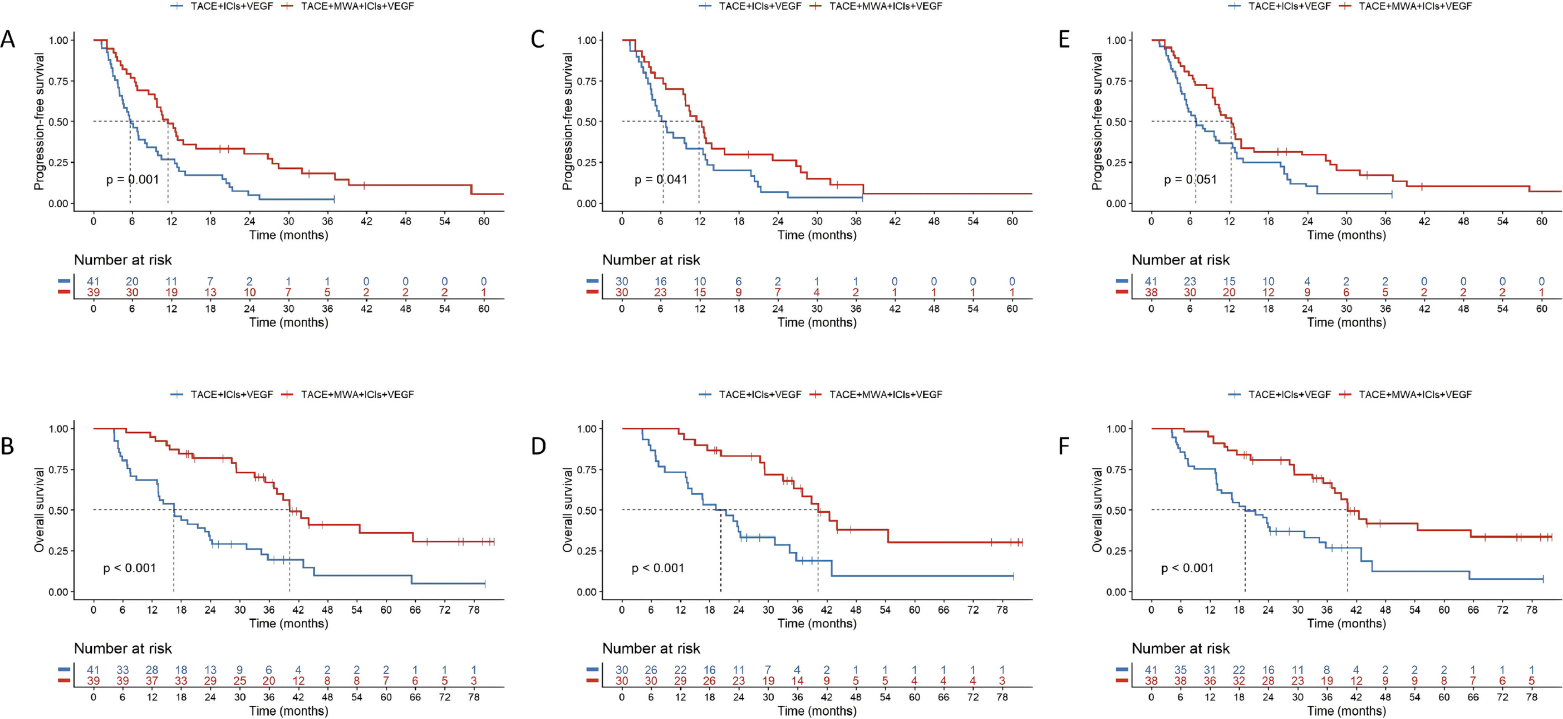
Kaplan–Meier analysis of PFS and OS between the study and control groups. Log-rank P values are shown in each panel. Primary cohort **(A, B)**, 1:1 PSM cohort **(C, D)**, and IPTW cohort **(E, F)**. OS, overall survival; PFS, progression-free survival; PSM, propensity score matching; sIPTW, stabilized inverse probability of treatment weighting.

The median follow-up time was 68.4 months (95% CI: 27.7–109.0). At the time of analysis, 21 patients (53.8%) in the study group and 35 patients (85.4%) in the control group had died. Overall survival (OS) was significantly longer in the study group compared with the control group [40.2 months (95%CI:34.302-46.165) vs. 16.5 months (95%CI:10.863-22.071), *P* < 0.001; HR: 0.307, 95% CI: 0.176–0.535]. Progression-free survival (PFS) was also significantly improved in the study group [11.4 months (95%CI:8.741-14.125) vs. 5.7 months (95%CI:3.617-7.716), *P* = 0.001; HR: 0.464, 95% CI: 0.286–0.751] (**Figures 2A, B**). One-, 3-, and 5-year OS rates, as well as 1-, 2-, and 3-year PFS rates, are summarized in **Table 2** (**Table 2**).

**Table 2:** Comparison of landmark PFS and OS rates between the two groups.

	Study group	Control group	*P* value
PFS			0.001
Median, months	11.4(8.741-14.125)	5.7(3.617-7.716)	
1-year, %	46.2%	24.4%	
2-year, %	27.3%	2.4%	
3-year, %	14.5%	NA	
OS			<0.001
Median, months	40.2(34.302-46.165)	16.5(10.863-22.071)	
1-year, %	92.3%	65.9%	
3-year, %	63.3%	19.5%	
5-year, %	30.6%	4.9%	

After PSM, the median OS was significantly longer in the study group than in the control group [40.2 months (95% CI: 35.233–NA) vs. 20.3 months (95% CI: 13.500–34.333), log-rank *P* < 0.001]. In contrast, the median PFS was 11.85 months (95% CI: 9.733–23.167) in the study group and 6.40 months (95% CI: 4.667–12.800) in the control group (log-rank *P* = 0.041)(**Figures 2C, D**). Overall, the direction and magnitude of the treatment effects after PSM were generally consistent with those observed in the original cohort. After sIPTW adjustment, the median OS was significantly longer in the study group than in the control group [42.5 months (95% CI: 35.230–65.430) vs. 21.4 months (95% CI: 13.500–31.300); weighted log-rank P < 0.001; adjusted HR for death, 0.37; 95% CI: 0.197–0.670]. The median PFS was 12.27 months (95% CI: 9.400–13.800) in the study group and 6.97 months (95% CI: 5.100–12.800) in the control group. Although the difference did not reach statistical significance (weighted log-rank P = 0.051), the study group demonstrated a trend toward improved PFS. The adjusted Cox model showed a trend toward reduced risk of progression or death (HR, 0.598; 95% CI: 0.343–1.044). (**Figures 2E, F**).

### Comparison of recurrence types

**Figure 3:**
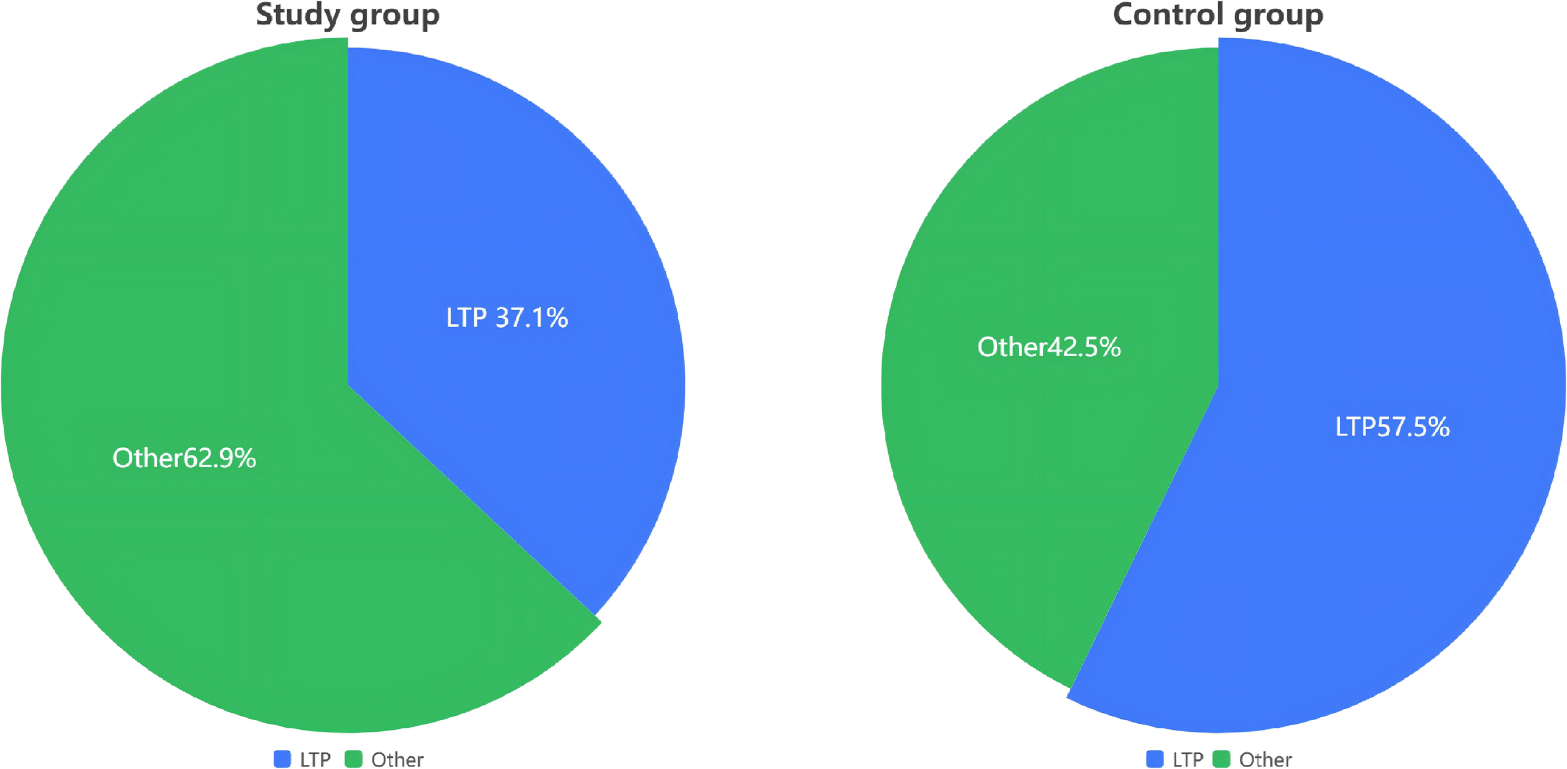
Patterns of recurrence in the study and control groups. LTP, local tumor progression.

A total of 35 patients in the study group and 40 patients in the control group experienced recurrence. Among them, 13 patients (37.1%) in the study group and 23 patients (57.5%) in the control group developed local recurrence. The local recurrence rate was significantly lower in the study group compared with the control group(**Figure 3**).

### Survival and recurrence related risk factors

Univariate Cox regression analysis showed that treatment regimen, AFP level, Child–Pugh score, cirrhosis, and BCLC stage were associated with overall survival (OS) (*P* < 0.05), while treatment regimen, AFP level, tumor number, and BCLC stage were associated with progression-free survival (PFS) (*P* < 0.05). These variables were subsequently included in the multivariate analysis.

Combination therapy was identified as an independent protective factor for PFS (HR = 0.483, 95%CI:0.295-0.791, *P* = 0.004), whereas AFP ≥200 ng/mL was an independent risk factor for PFS (HR = 2.131, 95%CI:1.285-3.534, *P* = 0.003). Combination therapy was also an independent protective factor for OS (HR = 0.351, 95%CI:0.199-0.617, *P* < 0.001) (**Table 3**).

**Table 3:** Univariate and multivariate Cox regression analyses of factors associated with PFS and OS.

Factor	PFS						OS					
	Univariate analysis			Multivariate analysis			Univariate analysis			Multivariate analysis		
	HR	95%CI	*P* value	HR	95%CI	*P* value	HR	95%CI	*P* value	HR	95%CI	*P* value
Group	**0.476**	**0.295–0.768**	**0.002**	**0.483**	0.295-0.791	0.004	**0.314**	**0.180–0.546**	**<0.001**	**0.351**	**0.199-0.617**	**<0.001**
Age	1.115	0.700–1.777	0.647				1.028	0.607–1.743	0.918			
Sex	0.894	0.523–1.528	0.681				1.008	0.539–1.886	0.98			
Hepatitis	1.139	0.717–1.809	0.580				0.905	0.535–1.531	0.087			
Cirrhosis	1.122	0.706–1.782	0.626				**1.924**	**1.103–3.3456**	**0.021**	1.566	0.841-2.915	0.157
AFP	**1.920**	**1.191–3.096**	**0.007**	**2.131**	**1.285-3.534**	**0.003**	**1.709**	**1.009–2.894**	**0.046**	1.469	0.847-2.547	0.171
Tumor Diameter	0.654	0.412–1.039	0.072				0.932	0.549–1.582	0.795			
Tumor Number	**1.616**	**1.010–2.587**	**0.045**	1.329	0.611-2.893	0.473	1.606	0.934–2.761	0.087			
Child-pugh	1.261	0.677~2.348	0.464				**2.032**	**1.047–3.943**	**0.036**	1.740	0.953-3.175	0.071
BCLC	**1.847**	**1.120–3.048**	**0.016**	1.680	0.745-3.790	0.211	1.654	0.914–2.994	0.097			

Bold indicates significance in the multivariate COX regression analysis.

BCLC, Barcelona Clinic Liver Cancer.

### Safety analysis

A total of 17 categories of treatment-related adverse events (AEs) were recorded. The incidence of any-grade AEs was 100% in both groups. Grade 3–4 AEs occurred in 13 patients (33.3%) in the study group and 10 patients (24.4%) in the control group. Locoregional treatment-related adverse reactions mainly included abdominal pain, fever, and hepatic dysfunction, all of which were transient and resolved after symptomatic treatment without delaying subsequent systemic therapy. Systemic therapy-related adverse events were grade 1–2 only, with no grade ≥3 events or permanent treatment discontinuation. No significant differences were observed between the two groups in the incidence of any-grade or grade 3–4 AEs (*P* > 0.05). The most common AEs in each group are summarized in **Table 4** (**Table 4**).

**Table 4:** Adverse events.

Adverse events	Any Grade		Grade 3-4	
	Study group(n=39)	Conrol group(n=41)	Study group(n=39)	Conrol group(n=41)
Abdominal pain	22(56.41)	18(43.90)	5(12.82)	2(4.88)
Fever	16(41.03)	21(51.22)	0(0.00)	0(0.00)
Nausea	14(35.90)	12(29.27)	0(0.00)	0(0.00)
Vomiting	16(41.03)	13(31.71)	0(0.00)	0(0.00)
Fatigue	9(23.08)	14(34.15)	0(0.00)	0(0.00)
Hypertension	14(35.90)	16(39.02)	0(0.00)	0(0.00)
Diarrhea	6(15.38)	3(7.32)	0(0.00)	0(0.00)
Decreased appetite	11(28.21)	8(19.51)	0(0.00)	0(0.00)
Rash	10(25.64)	11(26.83)	0(0.00)	0(0.00)
Gastrointestinal bleeding	3(7.69)	6(14.63)	0(0.00)	0(0.00)
Pneumonia	6(15.38)	3(7.32)	0(0.00)	0(0.00)
Neutropenia	16(41.03)	11(26.83)	4(10.26)	2(4.88)
Thrombocytopenia	15(38.46)	15(36.59)	8(20.51)	8(19.51)
Elevated ALT	22(56.41)	26(63.41)	9(23.08)	6(14.63)
Elevated AST	28(71.79)	31(75.61)	11(28.21)	7(17.07)
Proteinuria	7(17.95)	10(24.39)	0(0.00)	0(0.00)
Hypothyroidism	7(17.95)	8(19.51)	0(0.00)	0(0.00)

AST, aspartate aminotransferase; ALT, alanine aminotransferase.

## Discussion

This real-world retrospective study demonstrates that TACE combined with MWA improves local tumor control, and that subsequent sequential immune checkpoint inhibitors and anti-VEGF/TKI therapy significantly reduce recurrence and improve long-term survival in patients with HCC beyond the up-to-7 criteria.

TACE is a standard treatment for intermediate-to-advanced HCC; however, in patients beyond the up-to-7 criteria, high tumor burden, complex arterial supply, and rich collateral circulation often limit complete embolization, resulting in suboptimal local control and poor long-term outcomes (17). In contrast, the combination of TACE and MWA improves ablation efficacy and local tumor control, thereby improving prognosis.In this study, TACE combined with MWA improved local curative efficacy, reduced local recurrence, and prolonged progression-free survival and overall survival. Evidence from previous studies suggests that TACE combined with MWA may exert synergistic antitumor effects through multiple mechanisms. The potential mechanisms may include the following: TACE reduces tumor blood supply and mitigates the heat-sink effect during MWA, thereby enhancing intratumoral thermal deposition, expanding the ablation zone, and ensuring adequate safety margins (18, 19). In addition, for residual hypovascular lesions after TACE, including peripheral viable tumor tissue and incompletely embolized areas, MWA serves as an effective complementary ablative strategy. Through multi-point and multi-zone ablation, it further improves tumor eradication, particularly in large tumors with heterogeneous necrosis (20–22). Moreover, the combination of TACE and MWA may reduce tumor burden and reshape the tumor immune microenvironment, thereby creating more favorable conditions for subsequent systemic therapy (23, 24).

Systemic therapy following locoregional curative treatment has not yet been standardized. However, multiple studies have demonstrated favorable efficacy and safety of immune checkpoint inhibitors combined with anti-VEGF/tyrosine kinase inhibitors.Previous studies have suggested that TACE induces a hypoxic tumor microenvironment, leading to VEGF upregulation and an immunosuppressive microenvironment (25, 26). Anti-VEGF therapy may restore antitumor immunity and potentially enhance the efficacy of ICIs (26, 27). Meanwhile, MWA not only directly ablates tumors but may also promote antigen release, increase local immune cell infiltration, and potentially synergize with immunotherapy. Therefore, TACE combined with MWA may enhance local tumor control and potentially act synergistically with ICIs and anti-VEGF/TKI therapy (28–30). In the present study, patients exhibited high tumor burden, and a subset also had non-main portal vein tumor thrombosis and extrahepatic lymph node metastasis. For these patients, local therapy alone is often insufficient for adequate disease control. Therefore, sequential systemic therapy after locoregional treatment was implemented to enhance tumor control and improve management of vascular invasion and distant metastasis.Through these synergistic mechanisms, the combined local and systemic strategy may delay disease progression, translate into longer progression-free survival, and ultimately improve overall survival. In this study, combination therapy significantly prolonged both progression-free survival and overall survival, and multivariate Cox regression further confirmed it as an independent protective factor for both endpoints.Compared with the CHANCE2005 and EMERALD-1 trials, our cohort included patients with more advanced disease, as reflected by the poorer outcomes in the control group. Nevertheless, progression-free and overall survival in the treatment group were comparable to those reported in these prospective studies, which may be partly attributed to the additional immunomodulatory effects of MWA.In terms of safety, sequential targeted immunotherapy following TACE combined with MWA was well tolerated in patients with HCC beyond the up-to-7 criteria. No treatment-related deaths were observed. The most common grade 3–4 adverse events were transient liver injury and abdominal pain related to locoregional therapy, which did not delay subsequent systemic treatment. Overall, the safety profile was manageable.

This study has several limitations. First, its retrospective single-center design, small sample size, and non-randomized treatment allocation may have introduced selection bias and residual confounding, which could not be completely eliminated despite PSM and sIPTW analyses. Second, although systemic therapy initiation was used as the unified T0 to minimize potential immortal time bias, prospective validation is needed. In addition, heterogeneity in systemic treatment regimens and the lack of immune biomarker analyses may limit the interpretation and generalizability of the findings. Further multicenter prospective studies are warranted to validate this treatment strategy.

## Conclusion

In conclusion, for patients with hepatocellular carcinoma beyond the up-to-7 criteria, TACE combined with microwave ablation followed by targeted therapy and immunotherapy prolongs progression-free survival and overall survival compared with TACE combined with targeted therapy and immunotherapy alone. This multimodal strategy may represent a promising treatment option for patients with high tumor burden.

## Data Availability

The original contributions presented in the study are included in the article/**Supplementary Material**. Further inquiries can be directed to the corresponding authors.
