# Supplementary material for: Efficacy and safety of TACE combined with MWA followed by immune checkpoint inhibitors and anti-VEGF/tyrosine kinase inhibitors in hepatocellular carcinoma beyond the up-to-7
Source: Front Immunol. 2026 Jul 28;17:1885307. doi: 10.3389/fimmu.2026.1885307 (PMC13457286; doi:10.3389/fimmu.2026.1885307)
Supplement: Supplementary file 1 [file Table1.docx]

| **Agents** | **Targets** | **Administration** |
| --- | --- | --- |
| **Immune Checkpoints Inhibitors** | | |
| Sintilimab, Tyvyt®, Innovent Biologics, Inc., Suzhou, China | PD-1 | 200 mg once every 3 weeks, intravenous infusion |
| Camrelizumab, AiRuiKa®, Jiangsu Hengrui Medicine Co. Ltd, Suzhou, China |  | 200 mg once every 3 weeks, intravenous infusion |
| Tislelizumab, Baize’an®, BeiGene Ltd., Beijing, China |  | 200 mg once every 3 weeks, intravenous infusion |
| **Anti-VEGF antibody/ tyrosine kinase inhibitors** | | |
| Lenvatinib, Lenvanix®, Eisai Inc., Japan | VEGFR1–VEGFR3, PDGFR, FGFR1–FGFR4, RET | 8 mg QD (for bodyweight <60 kg) or 12mg QD (for bodyweight ≥60 kg) |
| Bevacizumab, Avastin®, F. Hoffmann-La Roche AG, Basel, Switzerland | VEGFA | 15 mg/kg once every 3 weeks, intravenous infusion |
| Sorafenib, Nexavar®, Bayer AG Kaiser-Wilhelm-Allee, Leverkusen, Germany | VEGFR1–VEGFR3,  PDGFR, RAF kinase, KIT receptor | 400 mg BID |
